# Finite Element Analysis of the Mechanical Performance of Non-Restorable Crownless Primary Molars Restored with Intracoronal Core-Supported Crowns: A Proposed Treatment Alternative to Extraction for Severe Early Childhood Caries

**DOI:** 10.3390/jcm12051872

**Published:** 2023-02-27

**Authors:** Kunyawan Thaungwilai, Yanee Tantilertanant, Weerachai Singhatanadgit, Pairod Singhatanadgid

**Affiliations:** 1 Composite Structures Research Unit, Department of Mechanical Engineering, Faculty of Engineering, Chulalongkorn University, Phyathai Road, Patumwan, Bangkok 10330, Thailand; 2 Faculty of Dentistry, Chulalongkorn University, Phyathai Road, Patumwan, Bangkok 10330, Thailand; 3 Faculty of Dentistry and Research Unit in Mineralized Tissue Reconstruction, Thammasat University (Rangsit Campus), Paholyothin Road, Klong Luang, Pathumthani 12121, Thailand

**Keywords:** early childhood caries, core build-up, stainless steel crown, crownless primary molars, finite element analysis, fatigue life

## Abstract

Early childhood caries (ECC) involve extensive coronal tooth structure loss, and tooth reconstruction remains highly challenging. To fulfill preclinical assessment, the present study investigated the biomechanics of non-restorable crownless primary molars that were restored by stainless steel crowns (SSC) using different composite core build-up materials. Computer-aided design-integrated 3D finite element and modified Goodman fatigue analyses were performed to determine stress distribution, risk of failure, fatigue life and dentine–material interfacial strength for the restored crownless primary molars. A dual-cured resin composite (MultiCore Flow), a light-cured bulk-fill resin composite (Filtek Bulk Fill posterior), a resin-modified glass-ionomer cement (Fuji II LC) and a nano-filled resin-modified glass-ionomer cement (NRMGIC; Ketac N100) were used as core build-up composite materials in the simulated models. The finite element analysis showed that types of core build-up materials affected the maximum von Mises stress only in the core materials (*p*-value = 0.0339). NRMGIC demonstrated the lowest von Mises stresses and revealed the highest minimum safety factor. The weakest sites were along the central grooves regardless of type of material, and the ratio of shear bond strength to maximum shear stress at the core–dentine interface of the NRMGIC group was lowest among the tested composite cores. However, all groups provided lifetime longevity from the fatigue analysis. In conclusion, core build-up materials differentially influenced the von Mises stress (magnitude and distribution) and the safety factor in crownless primary molars restored with core-supported SSC. However, all materials and the remaining dentine of crownless primary molars provided lifetime longevity. The reconstruction by core-supported SSC, as an alternative to tooth extraction, may successfully restore non-restorable crownless primary molars without unfavorable failures throughout their lifespan. Further clinical studies are required to evaluate the clinical performance and suitability of this proposed method.

## 1. Introduction

Dental caries of primary dentition is one of the most important children’s health problems worldwide [1]. Early childhood caries (ECC) is an important health problem in children and involves extensive coronal tooth structure loss [2]. ECC negatively influences both the child’s dental health and his/her general health [3]. An advanced stage of ECC causes severe pain, poor oral health-related quality of life, systemic infection, and eventually, premature tooth loss, potentially influencing children’s craniofacial development and nutritional status. Currently, a common treatment for a severely damaged non-restorable primary molar, e.g., a crownless molar, is extraction, which may result in impaired masticatory and phonetic efficiency, loss of vertical dimension, development of parafunctional habits, including tongue thrusting, malocclusion and space loss [3]. An easy-to-perform restorative technique that provides efficient, durable and functional restorations would prevent severely decayed primary teeth from an early loss, for example, direct resin composite restoration and stainless steel crown (SSC). While the longevity of posterior resin composite in patients with early childhood caries, reported in a retrospective study, was markedly influenced by patients’ health status [4], the application of SSC has been highly cost-effective in the treatment of extensive carious lesions in primary teeth. SSC has conquered other restorative materials in pediatric dentistry due to its durability and pliability [5]. A combined intracoronal core with SSC is a promising new treatment method that significantly strengthens crownless primary molars [6]. However, the durability of such a new method remains unknown.

Our previous study suggests that incompatibility in the mechanical properties between a core build-up material and dentine may increase the susceptibility of fracture at delicate dentine furcation under high force loading by initiating stress concentration at the thin furcation of the deciduous molar [6]. A stiffer luting material also increased stress concentration in tooth structure and reduced SSC stresses and deformations [7]. Various core build-up materials have been introduced, but whether these different core build-up materials have any effect on biomechanics, which are important to the overall treatment outcome, of the core-supported SSC remains unknown.

An in vivo study to investigate long-term performance, i.e., up to 8–9 years before the permanent tooth eruption, of this treatment using different core build-up materials is not practical. Prediction of its long-term durability using computational and numerical models is thus advantageous. These have vastly benefited from integrating finite element analysis (FEA) in the new treatment technique development process. FEA is a numerical method that solves boundary value problems in many medical applications. The FEA is a versatile numerical method because it applies to problems with a complex domain. For example, to determine the stress distribution in a solid body using the analytical method, where a closed-form solution is obtained, the problem’s domain is simple and must be able to describe in the form of algebraic equations. FEA has been employed in the dental field in several aspects. This typically translates toward verifying treatment performance in a virtual domain representative of its planned real-life application for dental treatment protocol development. Such an analysis helps indicate treatment performance and provides educated recommendations for improvement and optimization within a short time. Integrating the FEA into treatment protocol development may reduce costs over the development cycle. Such savings come to fruition by tentatively speeding up the process and reducing bench-testing iterations.

To fulfill preclinical assessment before testing in clinical trials, the present study investigated the biomechanics of crownless primary molars that were restored by SSC using different composite core build-up composite materials. Since we have previously validated that our analyses derived from defined FEA correlate well with fracture resistance and fracture characteristic experiments in vitro [6], the FEA using nonlinear static analysis and fatigue analysis was adopted in this study. Stress distribution and risk of failure in different compartments within the restorations and tooth structures were investigated. The fatigue life of the remaining dentine and interfacial stress between core build-up materials and dentine were also determined. The null hypothesis of this study proposed that no differences in the mechanical performance parameters existed between crownless primary molars that were restored with SSC using different composite core build-up materials.

## 2. Materials and Methods

Finite element modeling of the restored molars is presented in this section. In this study, FEA was employed to investigate the stress distribution, mechanical strength, and fatigue life of combined intracoronal core/SSC-restored crownless primary (complete coronal tooth structure loss) using four core build-up materials. A study on the relationship between PDL thickness and stress distribution is then explained to determine the appropriate modeling of the specimens. Finally, the methods to obtain stress distribution, safety factor and shear stress between the core material and dentine of the finite element model of restored molars using different core materials are given.

### 2.1. Finite Element Modeling and Analyses

**Figure 1:**
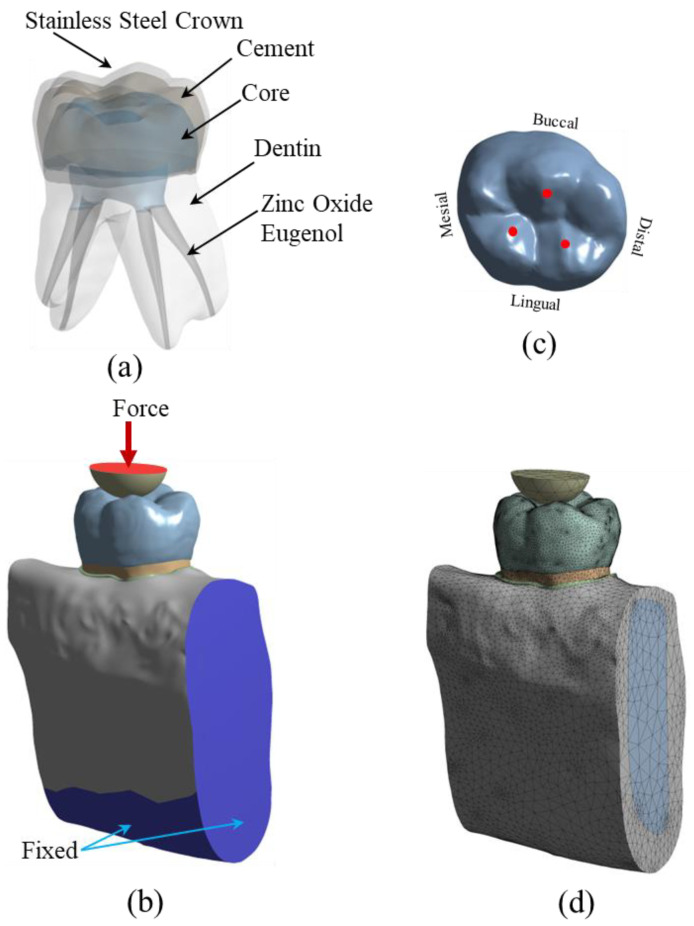
The model used in FEA. (**a**) 3D solid model of the restored tooth; (**b**) boundary and load conditions; the arrow represents applied vertical force; the blue areas were fixed in the FEA; (**c**) occlusal contact areas represented by red dots; and (**d**) meshed model using tetrahedron elements.

The stress analysis of a group of restored teeth with core build-up and SSC was performed using FEA. The core build-up was composed of four different types of materials, which include: (a) Dual-cured resin composite, DCRC (MultiCore Flow; Ivoclar Vivadent, Schaan, Liechtenstein), (b) Light-cured bulk-fill resin composite, LCRC (Filtek Bulk Fill posterior; 3M ESPE, St. Paul, MN, USA), (c) Resin-modified glass-ionomer cement, RMGIC, (Fuji II LC, GC, Tokyo, Japan), and (d) Nano-filled RMGIC, NRMGIC, (Ketac N100; 3M ESPE, Seefeld, Germany). Finite element modeling of the specimens was created from a solid model of a deciduous mandibular molar. A solid model of a sound tooth and jawbone was generated from the 3D CT-scanned image obtained from the Accuitomo 170 3D cone-beam computed tomography (CBCT) unit (J. Morita Mfg. Corp., Kyoto, Japan). The use of human tissue was approved by the Human Research Ethics Committee of Thammasat University No. 3 (COE No. 074/2560). Some adjustments on the scanned 3D image using the computer-aided design (CAD) software, CATIA v.5 (Dassault Systèmes, Vélizy-Villacoublay, France) and Blender software (https://www.blender.org (accessed on 1 March 2022)) were required to repair the incomplete data of the scanned images and to add some absent tissues such as the periodontal ligament (PDL) which were not presented in the CT-scanned image of the tooth. The software was also used to adjust and clarify the boundary between the cancellous and jawbone’s cortical bone. The solid model of the tooth was then placed in the jawbone. The solid model of a sound tooth is composed of enamel, dentine, dental pulp, PDL, cancellous bone and cortical bone, respectively. The sound tooth solid model was then modified to simulate the restoration with a combined intracoronal core and SSC. The components of the restored tooth include SSC, cement, core build-up material, zinc oxide eugenol, and the lower part of the remaining dentine, as shown in Figure 1a. The external size and shape of the SSC in the restored model were identical to the counterpart enamel in the original sound tooth. Contacts between each component of the model were set to simulate the mechanical behavior of the model. Contacts between some surfaces of the restored model were better modeled as “frictional.” For this type of contact, surfaces can slide relative to each other if shear stress at the contact exceeds a pre-defined value. Limiting shear stress is governed by the value of the coefficient of friction (*µ*) between both surfaces. In this study, frictional contacts in the restored model were contacts between (a) SSC and cement (*µ* = 0.2), (b) cement and core materials (*µ* = 0.2), and (c) zinc oxide eugenol and other components (core material, dentine and PDL, *µ* = 0.1). Contacts between other surfaces of the restored model were bonded.

To simulate the applied load during the chewing process, the restored tooth model was loaded with compressive force via a half-elastic sphere, as shown in Figure 1b. A constant vertical compressive load was applied to the top surface of the half sphere. There were three contact points between the half sphere and the SSC of the restored model, as shown in Figure 1c. A study by Rentes et al. [8] suggested that the biting forces in children are in the range of 161–330 N. An applied force of 200 N was used to simulate children’s masticatory force in the study by Pan et al. [9]. In this study, the applied force to the half sphere was set to be 100 N and 300 N. The applied force of 100 N was employed to simulate normal chewing behavior, while the 300 N force was used to simulate extreme chewing conditions, such as teeth grinding or bruxism [10,11,12,13]. The mechanical properties of the material of the half sphere were set to be identical to those of the enamel to simulate the chewing phenomenon. Besides applied compressive load on the half sphere, the model’s boundary conditions included fixed support on the bottom surface and two cross-sectional areas of the jawbone. In the FEA, the solid model of restored teeth was converted to a finite element model and analyzed using a commercial FEA software (ANSYS v.18; ANSYS Inc., Canonsburg, PA, USA). The meshing of the finite element model was performed using a nonlinear mechanical shape-checking function in ANSYS. The nonlinear meshing function allows a higher amount of mesh on the surface with higher curvature. The tetrahedron element with a maximum element size of 1 mm was utilized in this study. A total of 999,289 elements and 1,546,370 nodes were used in the model. A convergence study was performed to ensure that the obtained solutions were converged, i.e., mesh-independent. A finite element model of the restored tooth with typical meshing is presented in Figure 1d. Material properties, i.e., elastic modulus and Poisson’s ratio of the tooth components and restored materials, are presented in Table 1. All materials are assumed to be linear elastic and isotropic. In this study, both static stress analysis and fatigue analysis were performed. Static stress analysis returned stress distribution in all components of the model. The effect of different core build-up materials on stress distribution in each model component was investigated. Safety factors concerning the strength of each component were determined. The models were also examined for fatigue life using the fatigue analysis procedure. The model was simulated to be under cyclic loading, while induced stress in the model was determined and compared to the S-N curve of materials. Goodman’s mean stress correction theory was selected in this study.

### 2.2. Periodontal Ligament (PDL) Modeling

**Figure 2:**
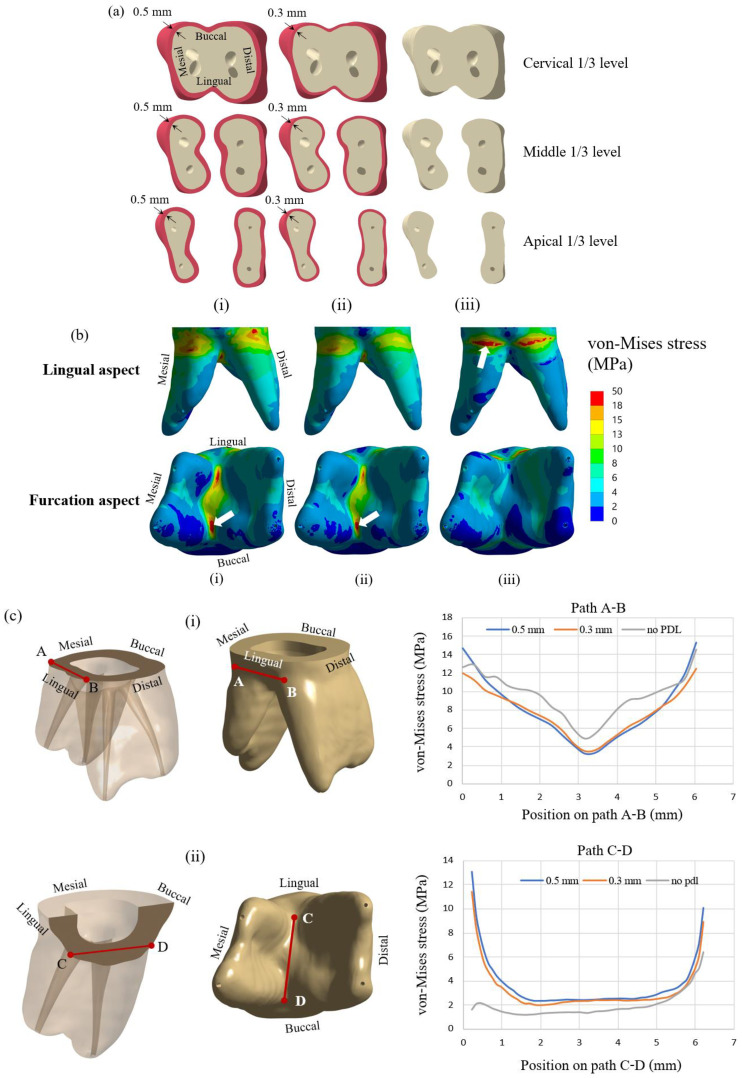

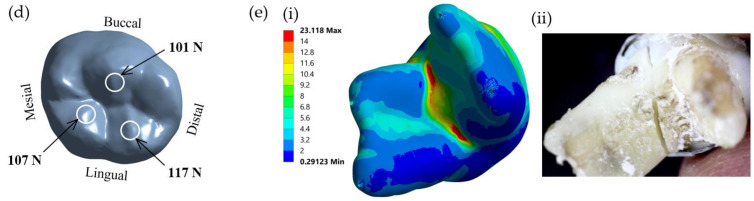
Simulated models using the DCRC core build-up material with 3 different PDL thicknesses, (**i**) PDL thickness = 0.5 mm; (**ii**) PDL thickness = 0.3 mm; (**iii**) No PDL, are shown in (**a**). In (**b**), distribution of von Mises stress in dentine with PDL thickness of (**i**) 0.5 mm (Maximum von Mises stress = 27.512 MPa); (**ii**) 0.3 mm (Maximum von Mises stress = 22.838 MPa); (**iii**) No PDL (Maximum von Mises stress = 45.149 MPa) following an occlusal load of 300 N is shown. Locations of maximum von Mises stress are indicated by white block arrows. The distribution of von Mises stress with and without PDL on (**i**) path A-B, and (**ii**) path C-D is shown in (**c**). In (**d**), contact forces at the occlusal contact areas are shown. Validation of the finite element method (**e**) was performed, and the von Mises stress distribution from the finite element method (**i**) corresponded to the fracture pattern from the in vitro experiment (**ii**).

PDL is a fibrous tissue that connects the tooth to the alveolar bone. The presence of the PDL allows the tooth structure to move relative to the bone slightly. It also prevents any direct contact between the tooth structure and the bone. Typically, the thickness of PDL is in the range of 0.15 to 0.38 mm [25], with a relatively low elastic modulus compared to other tooth components. In some studies, PDL was neglected in the finite element model [26,27,28,29], while it was included in some studies [30,31]. So, the effect of including PDL in the finite element model was investigated in the first part of the study. Finite element models of the teeth with PDL thicknesses of 0.3 and 0.5 mm and without PDL were prepared and analyzed for stress distribution. Cross-sections of the tooth model with PDL thickness of 0.5 mm and 0.3 mm and the model without PDL are presented in Figure 2a(i–iii), respectively. Three cross-sectional planes at three levels of dentine, i.e., cervical 1/3, middle 1/3, and apical 1/3 are shown in the figure. The elliptical holes shown in the figure are parts of the dental pulp. Dentine and other tooth components’ dimensions are identical for all three models. All models were subjected to an applied compressive load of 300 N on the half sphere, while stress distribution in the dentine of all three models was examined.

The FEA model used in this study was also validated with an experimental study. Test specimens were prepared from extracted primary mandibular molars. The present study was approved by the Human Research Ethics Committee of Thammasat University No. 3 (COE No. 074/2560). The samples were prepared using dual-cured resin composite core build-up materials and SSCs and loaded until failure. The experiment was carried out on a universal testing machine (Shimadzu, Kyoto, Japan) according to the procedures used in our previous study [6]. The failure patterns of the samples were examined and compared with the result of the finite element method. In the finite element investigation, a model of the restored molar was loaded with a force of 300 N, as described in the previous section. The von Mises stress distribution of the loaded model was plotted and analyzed. The region of maximum stress distribution of the FEA, which indicated the failure pattern of the sample, was compared with the failure pattern of the experiment.

### 2.3. Assessment of Safety Factor

This study obtained von Mises stresses in core build-up materials using FEM. The failure of core materials cannot be considered directly from the apparent stresses since the strength of each material is different from the others. The yield strength of the material is usually considered from the material’s yield stress, which is the stress level at which the material begins to deform plastically. This parameter is a material’s property obtained from material testing. With the von Mises failure criterion, the material is considered as “failed” if the von Mises stress at any point is equal to or greater than the yield strength of the material. A basic parameter that can be used to indicate whether a loaded structure comes close to its strength is the safety factor, which is defined as the ratio of the strength of material to the stress induced in the structure, i.e.,

(1) $$\mathrm{Safety}\;\mathrm{Factor}=\frac{\sigma_{Y}}{\sigma_{von}}$$

In this study, the strength of materials is the yield stress of materials $\sigma_{Y}$, which is a material’s property, while stress induced in the structure is von Mises stress $\sigma_{von}$ in the structure determined from FEM. Typically, if the safety factor of a structure equals 1, the structure is subjected to maximum stress, and it is at the theoretical onset of failure. If the safety factor is higher than 1, no failure is predicted, and the structure can sustain the additional load. A system with a higher safety factor can withstand a higher additional load before failure. Thus, it implies that the structure with a higher safety factor is stronger than the one with a lower safety factor. In this study, the stress induced in each type of core build-up material was different since their modulus was different. Instead of comparing the induced stress in each core build-up material, the safety factor was determined and used to compare the strength of the restored material. A system with a higher safety factor was considered stronger since it could withstand the additional load.

### 2.4. Determination of Fatigue Life of Dentine

Besides stress analysis to determine the stress distribution and safety factor of the restorative primary molar, fatigue analysis was also performed to determine the fatigue life of the dentine under cyclic loading. Fatigue failure can occur, although stress in the component is lower than the strength of the materials. When a component is subjected to repeated loading above a specific level, fatigue failure might be observed after a particular number of loading cycles. This study employed a stress-based approach and an *S*-*N* curve of material [32]. *S*-*N* curve or stress-life curve indicates the relationship between the number of cycles to failure, *N_f_*, when the material is loaded under cyclic load with the stress amplitude at zero mean stress,$\sigma_{a}$. The relationship between stress amplitude and life can be written as [32,33].

(2) $$\sigma_{a}=\sigma_{f}{\left(2N_{f}\right)}^{b},$$

where $\sigma_{f}$ is the fatigue-strength coefficient and *b* is the fatigue-strength exponent. For human dentine, the fatigue-strength coefficient $\sigma_{f}$ equals 247 MPa, and fatigue strength exponent *b* equals −0.111. So, the stress-life curve of human dentine is written as follows:

(3) $$\sigma_{a}=228.7N_{f}^{-0.111}.$$

This stress-life curve is obtained from simple cyclic tests where a specimen is subjected to constant-amplitude stresses, and the number of load cycles to failure is achieved and plotted versus the stress level. In some cases, if the applied stress is sufficiently low, the material does not fail by fatigue, i.e., it can be repetitively loaded infinitely. The maximum stress level at which the material does not fail is called the fatigue limit or endurance limit. Usually, the *S*-*N* curve is rarely directly applied to problems with complicated states of stress. The effects of multiaxial stress and mean stress must be accounted for in practical problems, where a specimen is usually subjected to multiaxial stress with non-zero mean stress. So, in this study, von Mises stress and modified Goodman diagram were employed in the Ansys program. The fatigue life of the dentine in the restorative molar with different core build-up materials was obtained from the FEA.

### 2.5. Analysis of Interfacial Stress between Core Build-Up Materials and Dentine

In addition to the failure of dentine or core build-up materials, interfacial debonding between core materials and dentine is another failure mode. For this mode of failure, neither dentine nor core materials failed, but the interfacial bonding between both components is broken down, resulting in a loss of function. This study determined the maximum shear stress on the dentine at the core–dentine interface and compared it with the shear bond strength between core build-up materials and dentine. Theoretically, if shear stress on that surface is lower than shear bond strength, the interfacial contact between both components is intact, and the restoration is successful. On the other hand, the bonding is said to be failed if the shear stress on the surface induced by the applied load is higher than the shear bond strength of the core materials against dentine. Maximum shear stress for the case of an applied load of 300 N was considered in this study.

### 2.6. Statistical Analysis

Descriptive statistics were used in the present study. In addition, one-way ANOVA was used to analyze the effect of different core build-up materials on the maximum von Mises stress and the minimum safety factor. Data derived from two occlusal loading forces were used for the analyses (N = 2). A *p*-value less than 0.05 was considered to be statistically significant, in which case the null hypothesis was rejected.

## 3. Results

### 3.1. Simulation of PDL Tissue and Validation of the FEA Model

The first part of the study investigated simulated models with different PDL thicknesses. Figure 2b presents von Mises stress distribution in the lingual and furcation aspects in the dentine for all three cases. The maximum von Mises stress in dentine was located at the furcation for the models with PDL thicknesses of 0.5 and 0.3 mm, whereas that for the model with no PDL space was located at the cervical area approximating the CEJ of the tooth (white block arrows). For the cases of the model with PDL, the maximum von Mises stress was observed at furcation with values of 27.51 and 22.84 MPa for the models with PDL thicknesses of 0.5 mm and 0.3 mm, respectively. On the other hand, the maximum von Mises stress for the model without PDL was 45.15 MPa and was observed in the cervical area. To examine stress inside the dentine, von Mises stresses on two imaginary lines, i.e., line A-B and line C-D, inside the dentine are presented in Figure 2c. Lines A-B and C-D are in the vicinity of cervical and furcation aspects, respectively. Line A-B is located horizontally inside the dentine on the lingual aspect of the tooth. It is on the same level as the top surface of the cortical bone. Line C-D is also located inside the dentine but near the furcation. It is placed from the lingual side to the buccal side. Distributions of von Mises stress on both lines for cases with and without PDL are presented in the figure. Along path A-B, von Mises stresses that occurred in the model without PDL were generally higher than those of the other models, with the mid-cervical area showing the lowest von Mises stress of approximately 3–5 MPa in all three models. Approximately 12–15 MPa von Mises stresses occurred at the mesiolingual area and distolingual area of the lingual aspect (Figure 2c(i)), while much lower von Mises stresses (around 1–3 MPa) were observed at the cervical portion of the buccal aspect (data not shown). In contrast to path A-B, the model without PDL space showed lower von Mises stress at the furcation area along path C-D, especially at the lingual portion of the furcation (2 MPa vs. 11–13 MPa) (Figure 2c(ii)). In both areas, the stress distribution of the models with PDL is close to each other on both lines but fairly different from that of the model without PDL. Taken together, the results indicated that the presence of PDL tissue in the simulated model of a crownless primary posterior molar restored with core-supported SSC had a pronounced influence on the distribution and level of the von Mises stress following occlusal loading force. Therefore, a model with a PDL thickness of 0.3 mm, which closely resembles the thickness of normal PDL space, was used in all subsequent experiments.

A comparison of the von Mises stress distribution of the FEA model and the fracture pattern of the in vitro experiment on the restored primary mandibular molars is presented in Figure 2e. The maximum von Mises stress from the FEA was concentrated in the root furcation region (Figure 2e(i)). The corresponding fracture failure pattern obtained from the experimental samples was also observed, i.e., at the molar furcation along with the bucco-lingual direction, and a representative fractured experimental molar is shown in Figure 2e(ii).

### 3.2. Stress Distribution in Crownless Primary Posterior Teeth

To investigate the influence of different core build-up materials used in core-supported SSC for restoration of crownless teeth on stress distribution, safety factor, and fatigue life of the dentine, crownless primary posterior teeth restored with core-supported SSC were loaded with two different occlusal loading forces, i.e., 100 N and 300 N, which are in the range of normal physiologic chewing force and non-physiologic chewing force, such as those occurring during clenching in children [10,11,12,13]. Maximum von Mises stresses in the SSC, dentine, core material, PDL and bone of the models using different core build-up materials receiving 100 N and 300 N occlusal loading forces are summarized in Table 2 and Table 3, respectively. It was observed that stress induced in each core build-up material was different, while stresses in the other parts of the model, i.e., SSC, dentine, PDL and bone, were independent of core build-up material types. Moreover, types of core build-up material highly influenced stress values irrespective of occlusal loading forces. Maximum von Mises stresses in DCRC, LCBRC, RMGIC and NRMGIC were approximately 18.9, 18.6,16.5 and 10.6 MPa, respectively, for the applied load of 100 N (Table 2), and 22.5, 21.7, 19.3 and 12.5 MPa, respectively, for the applied load of 300 N (Table 3). The maximum von Mises stress in the model using NRMGIC was lower than those using the other core build-up materials (*p*-value = 0.0339). Comparing the von Mises stress between the models receiving 100 N and 300 N, two-fold increases in stress were found in the whole restored tooth and dentine, while three-fold increases were observed in the PDL and bone (Table 2 vs. Table 3). Interestingly, von Mises stress increased only 16–20% in core build-up materials when the applied occlusal force increased from 100 N to 300 N regardless of the types of core build-up material (Table 2 vs. Table 3).

**Figure 3:**
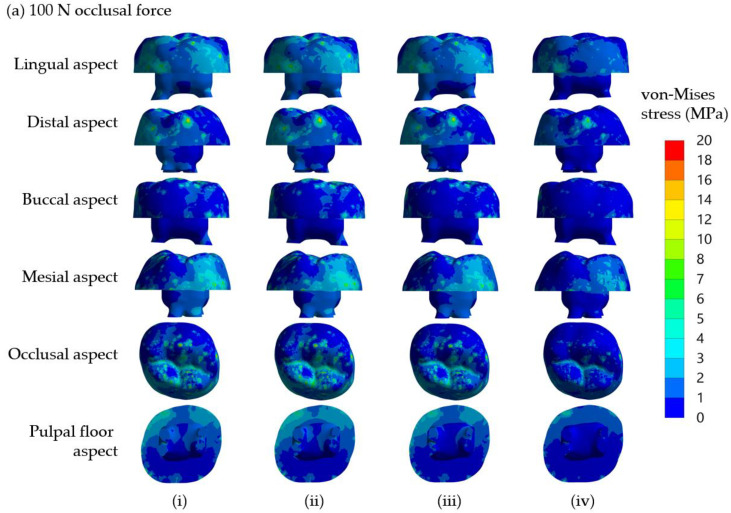

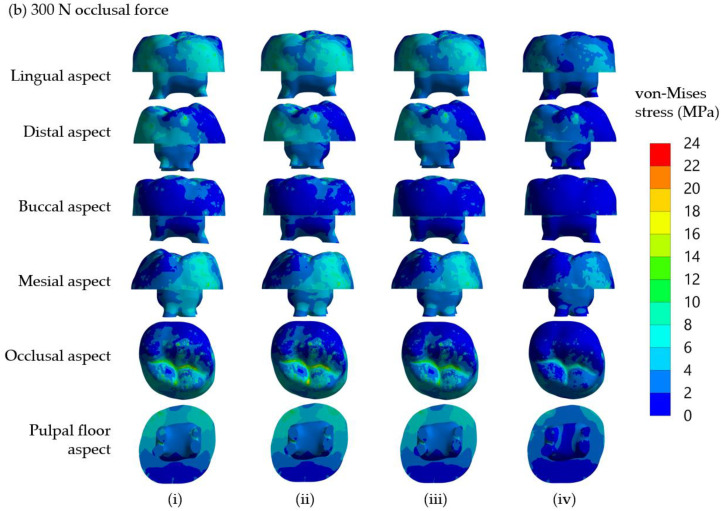
Distribution of von Mises stress in core materials of the models using four different core build-up materials, (**i**) DCRC, (**ii**) LCRC, (**iii**) RMGIC and (**iv**) NRMGIC, following 100 N and 300 N occlusal loading forces are shown in (**a**) and (**b**), respectively.

The distributions of von Mises stress in core materials, which varied among the models using four different core build-up materials, are demonstrated in Figure 3. In three models using the core build-up materials DCRC, LCRC and RMGIC, a similar distribution pattern of von Mises stress was observed, although generally higher stresses were found in the models receiving an occlusal loading force of 300 N compared with those receiving 100 N. Stresses less than 6 MPa, considered low stress, were observed only at the buccal aspect in all models receiving either 100 or 300 N loading force. In contrast to those three models, the model restored with NRMGIC possessed a much lesser distribution of von Mises stress higher than 6 MPa. This high stress is concentrated only along the central groove on the occlusal aspect of the core build-up material.

Taken together, the results indicated that the maximum von Mises stress and von Mises stress distribution in crownless primary posterior teeth restored with core-supported SSC were affected by the type of core build-up materials.

### 3.3. Safety Factor

The fracture resistance of a material depends on not only the stress presented in it but also its strength. Therefore, we determined the safety factor, which corresponds to fracture resistance of the models’ SSC, dentine and core build-up material using different core build-up materials under applied occlusal loading forces of 100 N and 300 N, and the results are summarized in Table 4 and Table 5. Thus, safety factors in DCRC, LCBRC, RMGIC and NRMGIC were approximately 2.7, 2.2,2.7 and 5.2, respectively, for the applied load of 100 N (Table 4), and 2.2, 1.9, 2.3 and 4.4, respectively, for the applied load of 300 N (Table 5). As with the maximum von Mises stress, different core build-up materials affected the safety factor that only occurred in the core build-up material regardless of the force loaded (*p*-value = 0.0054). It is noteworthy that due to the direct occlusal loading contact on the SSC surface, the safety factor of SSC in all models appeared lower than 1, suggesting that the SSC would be vulnerable to failure. Considering the safety factor within the core build-up material, the safety factor in the model using NRMGIC was approximately two folds higher than those using the other core build-up materials, regardless of the applied occlusal force. Comparing the safety factor between the models receiving 100 N and 300 N, two-fold decreases in safety were found in the SSC and dentine. In contrast, this decreased only 14–16% in core build-up materials when the applied occlusal force increased from 100 N to 300 N regardless of the type of core build-up material (Table 4 vs. Table 5). Taken together, the results suggested that the core build-up may be less sensitive to a change in occlusal force and that NRMGIC may be the most resistant core build-up material against occlusal force when combined with SSC in primary teeth.

**Figure 4:**
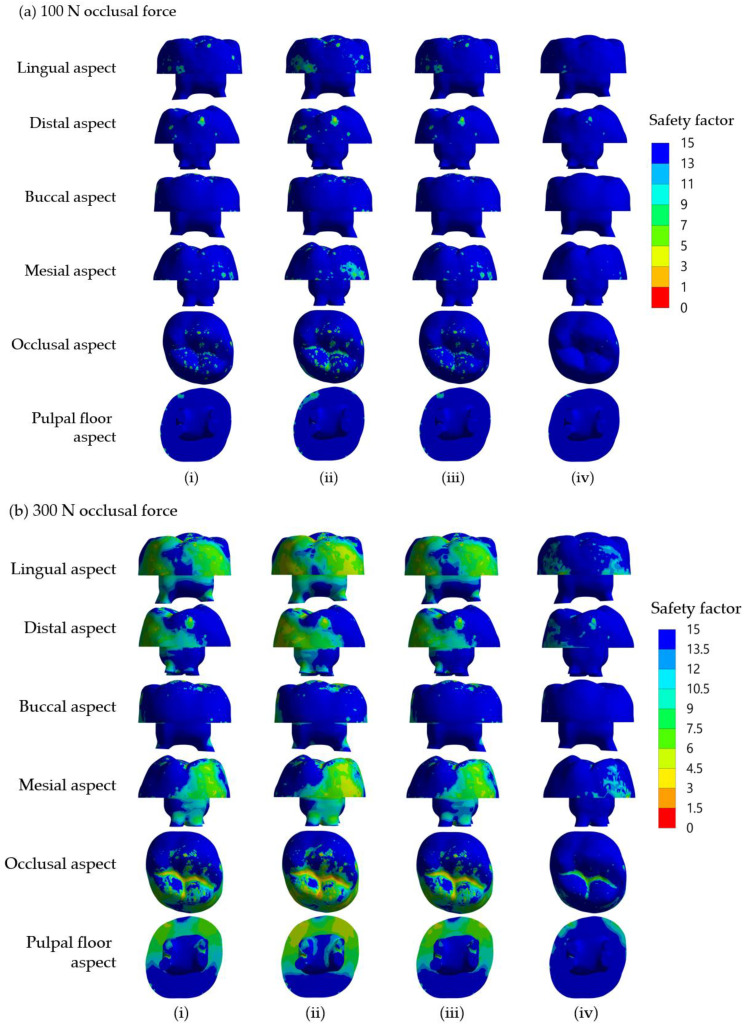
Distribution of safety factor in core materials of the simulated models using four different core build-up materials, (**i**) DCRC, (**ii**) LCRC, (**iii**) RMGIC and (**iv**) NRMGIC, following 100 N and 300 N occlusal loading forces are shown in (**a**) and (**b**), respectively.

Figure 4 shows the distributions of safety factor in core build-up materials, which only varied slightly among the models using the three different core build-up materials, DCRC, LCRC and RMGIC, regardless of the occlusal force applied. Despite the non-significant difference, NRMGIC showed the highest safety factors on all surfaces of the build-up core, which ranged between 13.5 and 15 compared to other tested core build-up materials. As expected, generally higher safety factors were found in the models receiving an occlusal loading force of 100 N (Figure 4a,b). The three models using three different core build-up materials, DCRC, LCRC and RMGIC, showed no difference in the distribution of safety factor on all surfaces, although generally higher safety factors were found in the models receiving an occlusal loading force of 100 N (Figure 4a) compared with those receiving 300 N (Figure 4b). Safety factors of less than 6 were observed on all surfaces except in the buccal aspect for the models receiving either 100 or 300 N loading force. The low safety factor appeared to concentrate along the central groove on the occlusal aspect of the core build-up material in all core build-up material groups, indicating the occlusal groove as the weakest point to be fractured.

### 3.4. Fatigue Life of the Dentine

Since the favorable failure of either SSC or build-up core within the restored crownless primary molars should be repairable, we thus focused on the determination of longevity of the remaining tooth structure. We carried out a fatigue analysis based on the von Mises stress and modified Goodman diagram to assess the fatigue life of the dentine in the four models. The minimum cycles to failure of dentine of the models using four different core build-up materials under 300 N occlusal loading force are presented in Table 6, which demonstrates that the dentine in the four models fractured following approximately 9 × 10^8^, 8 × 10^8^, 7 × 10^8^ and 4 × 10^8^ loading cycles, respectively. Although the dentine in the NRMGIC model appeared to be fractured faster than the others, all models seemed to provide lifetime longevity.

**Figure 5:**
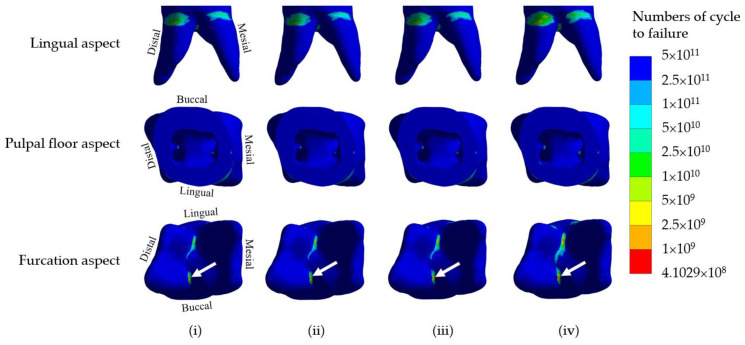
Distribution of fatigue life (numbers of cycle to failure) in the dentine of the simulated models using four different core build-up materials, (**i**) DCRC, (**ii**) LCRC, (**iii**) RMGIC and (**iv**) NRMGIC, receiving 300 N occlusal loading force. White arrows indicate the initiation site of failure.

The distribution of fatigue within the dentine of the simulated models using four different core build-up materials is shown in Figure 5. The initiation of the fracture where the shortest fatigue life is located occurred at the furcation area regardless of the type of material used for the core build-up. The fatigue life of the remaining dentine of crownless primary posterior teeth restored with core-supported SSC also provided lifetime longevity.

### 3.5. Core-Dentine Interfacial Stress

**Figure 6:**
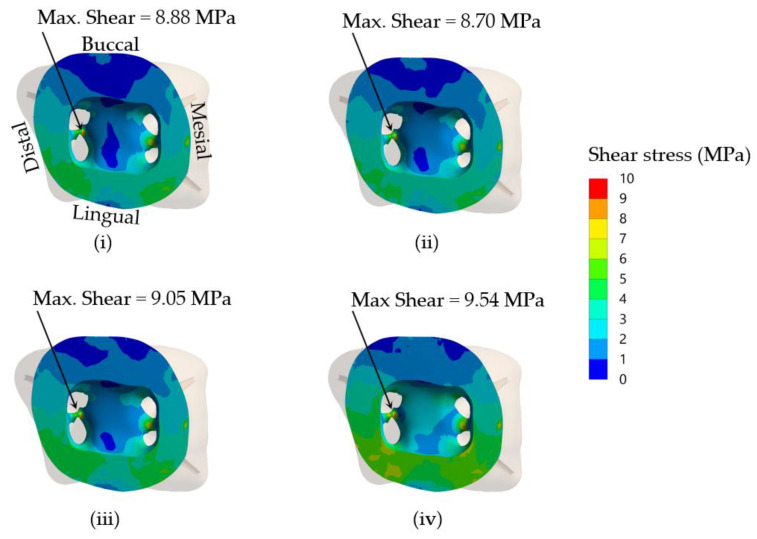
Distribution of shear stress in dentine at the core–dentine interface of the simulated models using four different core build-up materials, (**i**) DCRC, (**ii**) LCRC, (**iii**) RMGIC and (**iv**) NRMGIC, following 300 N occlusal loading force. The maximum shear stress values are also shown.

The results showed that maximum shear stresses that occurred at the core–dentine interface were approximately 8.9, 8.7, 9.1, and 9.5 MPa in the simulated models using DCRC, LCRC, RMGIC and NRMGIC, respectively (Figure 6). Considering previously reported maximum shear bond strengths against primary tooth dentine of a self-etch adhesive (used for DCRC and LCBRC core build-up materials), RMGIC and NRMGIC of approximately 11.3, 9.9 and 6.3 MPa, respectively, [34,35,36] the ratios of shear bond strength/maximum shear stress in dentine at the core–dentine interface of the simulated models using four different core build-up materials, DCRC, LCRC, RMGIC and NRMGIC, following 300 N occlusal loading force were 1.3, 1.3, 1.1 and 0.7, respectively (Table 7). This indicated that NRMGIC might be most vulnerable to dislodging from the pulpal dentine.

## 4. Discussion

The present study investigated the biomechanics of crownless primary molars restored by different core build-up materials combined with SSC using a computer-aided design-integrated 3D FEA. The null hypothesis of the present study was rejected. The results demonstrated that types of core build-up materials and occlusal loading differentially influenced the mechanical performance-related parameters in the SSC, core build-up materials, dentine, PDL and alveolar bone. The model in the present study included the PDL tissue to mimic a realistic situation. Our result demonstrated a difference in stress distribution in the presence of PDL tissue. The von Mises stress at the cervical area in the model without PDL was higher than that of the model with PDL. Stress distributions on the lingual and buccal sides of the models were also different for models with and without PDL. With an extremely low modulus of elasticity, PDL served as a force-absorbing cushion between the dentine and the cortical bone, facilitating the transmission of loading force. Therefore, the model with PDL observed a lower stress concentration on the dentine in the cervical area. Such stress was subsequently transferred to the furcation area and revealed higher stress concentration in that region. The change of stress value was more intense at PDL than in the restorative material and dentine following 300-N loading force, affirming the function of PDL as a masticatory apparatus to help distribute the stress to the alveolar bone [37]. A recent study by Maravic et al. [38] also demonstrated that the presence of PDL in the model influenced the result of stress distribution in both restorative materials and tooth structure. Moreover, finite element models with PDL were used in several studies that tested the stress and strength of various restorative materials, including glass ionomer cement and resin composite [39,40].

In the present study, the finite element investigation of the restored crownless primary molar was validated with an experimental in vitro fracture test. The region of maximum stress of the model simulated in the FEA was comparable to the fracture pattern of the experimental study. Thus, the finite element models and analyses in the present study were satisfactorily validated. However, future in vitro fracture experiments with specific composite materials used in the present study will unequivocally confirm their potential clinical application.

It is found that von Mises stress on the lingual side is higher than on the buccal side. This observation can be explained by considering the applied compressive force on the restored tooth model, as shown in Figure 2d. With the solid model of the restored first molar and a half sphere, the compressive forces were applied to three points of the SSC. From the distribution of contact force from the half sphere on the SSC, it was found that there were two forces of 107 N and 117 N on buccal incline planes of the mesiolingual and distolingual and a force of 101 N on the lingual incline plane of the distobuccal cusp (Figure 2d). As a result of the uneven contact forces on the lingual and buccal sides, the tooth was loaded on the lingual more than the buccal side. Therefore, the model tended to rotate from the buccal side to the lingual side; thus, the reaction force on the dentine is higher on the lingual side. This observation corresponds very well with the stress analysis from FEM, i.e., the von Mises stress on the lingual side of the cervical area is higher than that on the buccal side.

For SSC restorations in molar teeth, the most common etiology of ‘true’ failures is from mechanical reasons rather than the development of recurrent caries, and one of the primary mechanical causes appears to be occlusal perforation of the SSC [41]. The Roberts et al. study showed that the prevalence of SSC occlusal perforation was higher than cement failure [42]. In the present study, the maximum safety factors of the SSC in all groups were much lower than those of the dentine and core build-up materials under 100 N and 300 N occlusal loading forces (Table 4 and Table 5). It is thus very likely that the failure that occurred in the SSC will be the cause of the restoration failure. Clinically, occlusal surfaces of SSCs for primary molars display wear and subsequent perforation, which can be repaired using a glass ionomer cement or a packable composite resin [43]. Therefore, SSC perforation is possibly caused by high-stress concentration on the occlusal surface shown in the present models.

The von Mises stress changed differently, respective to the type of restorative materials. The results of the present study are in agreement with those of Conserva et al. [44] who suggest that a composite core with Young’s modulus similar to that of dentin is a material of choice for the reconstruction of endodontically treated teeth. Mechanical properties play a role in determining the risk of failure. Mechanical properties of a glass ionomer cement are markedly dependent on its compositions, including the amount and size of fillers, the integrity between glass filler and polymer matrix, modification of polyacrylic acid and microstructure defects [45,46]. Different compositions in each of the materials simulated as build-up cores in the present study affect Young’s modulus values and thus influence the maximum safety factor of the core build-up (Table 4 and Table 5). RMGIC consists of hydrophilic resin monomers (2-hydroxyethylmethacrylate (HEMA)) and a photo-initiator [47], while NRMGIC contains nano-sized filler and bioceramic particles added to RMGIC [48]. Such addition of nanofillers made the NRMGIC better resist the loading force and thus provided a higher safety factor than the RMGIC did; it also showed high clinical performance as a direct restoration in deciduous teeth [49]. However, our FEA revealed that both simulated core build-up materials for SSC restoration of crownless primary molars possessed lifetime longevity. Future clinical trials are undoubtedly required to support this.

Interfacial bond strength is a key factor influencing the overall mechanical properties of a bonded restoration by improving interlaminar adhesion, debonding resistance and fatigue resistance [50]. To obtain desired restoration outcome of using a core build-up and SSC placement in severely damaged primary molars, the interfacial bond strength should be high enough to resist the maximum shear stress within the dentine–core build-up material interface. Using a finite elemental method, it is possible to estimate the maximum interfacial stress and thus predict the longevity of the restoration. The ratios of shear bond strength/maximum shear stress in dentine at the core-dentine interface of NRMGIC were the lowest. This suggested that interfacial bond strength between NRMGIC and tooth dentine is the most vulnerable to occlusal force, and that a dislodged core build-up may cause the failure of the SSC restoration. NRMGIC may be unable to sufficiently transfer loading force through the core–dentine bonding interface due to its highest maximum shear stress and very low bond strength (Table 7). According to its lowest von Mises stress, NRMGIC-restored core under SSC probably contributed to a favorable failure following an occlusal loading. It is, however, not currently possible to predict the longevity of the interfacial bond of the simulated models. Future studies on the fatigue resistance of each bonding system needed to determine their estimated longevity are warranted.

Several factors influence the success of SSC restoration in clinical settings, including that for severely damaged primary molars. These include marginal adaptation, extension and proximal contacts of SSCs, and plaque and gingival bleeding at SSC [51]. A recent finite element study showed that a stiffer luting material used for SSC cementation increases stress concentration in tooth structure and reduces SSC stresses and deformations, while stresses within alveolar bone appear unchanged regardless of the cement type used [7]. We have previously shown that restoration of severely damaged primary molars with core build-up and SSC placement markedly increases fracture resistance. However, the failure mode seems unfavorable, as shown in the fractured root furcation [6]. However, the present FEA study suggests that all the core build-up materials used provide lifetime durability in humans, suggesting that restoration using a core build-up and SSC placement is a promising treatment option for severely damaged primary molars with excellent longevity and repairable SSC perforation if it happens. This corresponds to a previous clinical report on SSC’s very high overall success rate [51]. Since such treatment techniques are preferable in primary teeth which have a limited lifespan, balancing between reinforced remaining tooth structure and favorable failure should be carefully considered to select the proper choice of core build-up materials. Further clinical trials will ensure the success of this treatment modality for severely damaged primary molars in the real world.

Although restoration of crownless primary molars by a simple filling method may be technically easier and consumes shorter treatment time, the use of an intracoronal core-supported crown can provide much higher longevity and lower risk for secondary caries. Management of early childhood caries by multiple extractions can result in impaired masticatory and phonetic efficiency, loss of vertical dimension and development of parafunctional habits. We believe that when possible, the use of intracoronal core-supported crown is a good treatment alternative to multiple tooth extraction in children with severe early childhood caries.

The limitations of the present study include a lack of experimental in vitro cyclic fatigue testing, which should be considered for future studies to better simulate clinical scenarios [52]. The fatigue fracture resistance of each core build-up material and the durability testing of the bonding systems used can also help determine their estimated longevity.

## 5. Conclusions

The finite element method was utilized to investigate the mechanical behaviors of crownless primary molars restored with SSC supported by intracoronal core build-up using four different composite materials. Within the limitations of this investigation, the following conclusions can be drawn:

1. The presence of PDL noticeably affected stress distribution and maximum von Mises stress in crownless primary posterior teeth restored with core-supported SSC.

2. Different core build-up materials differentially influenced the magnitude and distribution of the von Mises stress and safety factor in crownless primary posterior teeth restored with core-supported SSC.

3. All the core build-up used in the present study and the remaining dentine in crownless primary posterior teeth restored with core-supported SSC possessed lifetime longevity.

The present study suggests that the reconstruction by core-supported SSC, as an alternative to tooth extraction, may successfully be used to restore non-restorable crownless primary molars without unfavorable failures throughout their lifespan. This offers dentists a feasible option for maintaining the functions of primary molars, which otherwise would have undergone extraction. Further clinical studies are required for clinical outcomes and suitability assessments of this proposed method in children experiencing severe early childhood caries.

## Figures and Tables

**Table 1:** Properties of tooth structures and dental restorative materials used in the study.

Tooth Structures/Materials	Elastic Modulus (MPa)	Poisson’s Ratio	Yield Strength (MPa)	References
Enamel	84,100	0.2	60	[14,15]
Dentine	18,600	0.31	86.8	[14,15]
Pulp	2	0.45	-	[16]
Periodontal ligament (PDL)	68.9	0.45	-	[16]
Cortical bone	13,700	0.3	-	[17]
Cancellous bone	1370	0.3	-	[17]
Zinc oxide eugenol [4]	2140	0.28	-	[18]
Stainless steel crown (SSC)	200,000	0.33	250	[19]
SSC Luting cement	10,860	0.3	45	[20]
Dual-cured resin composite (DCRC) (MultiCore Flow; Ivoclar Vivadent, Schaan, Liechtenstein)	16,000	0.26	50.6	[21,22]
Light-cured bulk-fill resin composite (LCRC) (Filtek Bulk Fill posterior; 3M ESPE, St. Paul, MN, USA)	13,460	0.18	41.1	[22,23]
Resin-modified glass ionomer (RMGIC) (Fuji II LC, GC, Tokyo, Japan)	10,860	0.3	45	[20,24]
Nano-filled RMGIC (NRMGIC) (Ketac N100; 3M ESPE, Seefeld, Germany)	4000	0.44	55	[24]

**Table 2:** Maximum von Mises stress (MPa) in the SSC, dentine, core material, PDL and bone of the models using different core build-up materials under 100 N occlusal loading force.

	DCRC	LCBRC	RMGIC	NRMGIC
SSC	607.76	606.70	605.85	598.40
Dentine	11.14	11.15	11.20	11.29
Core	18.93	18.57	16.51	10.59
PDL	1.43	1.43	1.42	1.39
Bone	6.93	6.93	6.92	6.89

**Table 3:** Maximum von Mises stress (MPa) in the SSC, dentine, core material, PDL and bone of the models using different core build-up materials under 300 N occlusal loading force.

	DCRC	LCBRC	RMGIC	NRMGIC
SSC	1346.20	1344.70	1342.40	1332.50
Dentine	23.11	23.49	23.76	25.30
Core	22.51	21.74	19.27	12.47
PDL	3.22	3.21	3.20	3.16
Bone	17.05	17.06	17.02	16.89

**Table 4:** Minimum safety factor of the SSC, dentine and core build-up material of the models using different core build-up materials under 100 N occlusal loading force.

	DCRC	LCBRC	RMGIC	NRMGIC
SSC	0.41	0.41	0.41	0.41
Dentine	7.79	7.78	7.74	7.68
Core	2.67	2.21	2.72	5.19

**Table 5:** Minimum safety factor of the SSC, dentine and core build-up material of the models using different core build-up materials under 300 N occlusal loading force.

	DCRC	LCBRC	RMGIC	NRMGIC
SSC	0.18	0.18	0.18	0.18
Dentine	3.75	3.69	3.65	3.43
Core	2.24	1.89	2.33	4.40

**Table 6:** Minimum numbers of cycles to failure of dentine of the models using different core build-up materials under 300 N occlusal loading force.

	DCRC	LCBRC	RMGIC	NRMGIC
Fatigue (cycles)	9.2628 × 10^8^	8.0174 × 10^8^	7.226 × 10^8^	4.1029 × 10^8^

**Table 7:** Maximum shear stress and shear bond strength/maximum shear stress ratios in dentine at the core–dentine interface of the simulated models using different core build-up materials under 300 N occlusal loading force.

	DCRC	LCRC	RMGIC	NRMGIC
Shear bond strength (MPa) *	11.3	11.3	9.9	6.3
Maximum shear stress (MPa)	8.9	8.7	9.1	9.5
Strength/stress ratio	1.3	1.3	1.1	0.7

* Previously reported maximum shear bond strength against primary dentine [34,35,36].

## Data Availability

Not applicable.
